# Federated asynchronous graph attention network with structural semantic embedding for multi-label graph classification

**DOI:** 10.1038/s41598-025-22500-6

**Published:** 2025-11-04

**Authors:** Xinwu Ji, Yijing Zhang, Kaihong Zheng, Hao Yang, Yuan Ai, Jingxu Yang

**Affiliations:** 1 China Southern Power Grid, Yunnan Power Grid Co., Ltd, Kunming, 650000 China; 2 China Southern Power Grid Digital Grid Group Co., Ltd., Guangzhou, 510700 China

**Keywords:** Federated learning, Multi-label learning, Graph attention neural network, Computational biology and bioinformatics, Mathematics and computing

## Abstract

Federated Learning (FL) provides a privacy-preserving framework for training graph neural networks (GNNs) in privacy-sensitive scenarios. However, traditional FL-GNN approaches often focus on addressing data distribution inconsistencies across clients from a purely data-centric viewpoint, overlooking the critical role of label semantics. Incorporating label semantic information, however, significantly enhances a model’s performance in multi-label classification tasks. Moreover, the performance of FL-GNNs is constrained by two key challenges related to heterogeneity: intra-client heterogeneity in graph representations and inter-client heterogeneity across distributed graphs. Unfortunately, few FL methods effectively handle discrepancies in both label distributions and graph heterogeneity across different clients. To address this gap, we introduce the federated asynchronous graph attention network with structural semantic embedding for multi-label classification (FasSGAT). FasSGAT primarily contains: (1) client-specific label semantic embedding modules that learn feature encodings from constructed label-semantic distribution graphs, (2) the integration of these encodings into the backbone multi-label classifier, along with specially designed structure-sensitive spectral features to mitigate client-side heterogeneity, and (3) a novel structure-sensitive asynchronous aggregation mechanism at the server level that uses the graph spectral features to construct a global model and address graph heterogeneity. Our experimental results on multi-label benchmarks show that FasSGAT outperforms traditional FL methods across various evaluation metrics.

## Introduction

Federated learning (FL), through frameworks like Federated Averaging (FedAvg)^1,2^, has enabled privacy-preserving collaborative training, yet it encounters significant challenges, particularly with data inconsistency arising from non-IID distributions across clients. These challenges intensify when applied to graph-structured data, where Graph Neural Networks (GNNs) are often employed to enhance classification performance by leveraging the graph structure to learn structural information. However, while FL provides a privacy-conscious alternative to centralized GNN training, it still struggles with addressing inconsistencies in data distribution across clients^3–6^. Most current FL-GNN frameworks focus primarily on data distribution discrepancies but fail to consider the different semantic information embedded in label distributions across different clients. This oversight prevents the model from fully capturing the underlying context and relationships within the data. In multi-label classification tasks, where data points can be linked to multiple labels, the lack of semantic alignment between the labels and data across clients can severely impair model performance^5^, particularly in clients with differing label distributions^7,8^. Furthermore, for graph classification, FL-GNN performance is hindered by two major types of heterogeneity: intra-client heterogeneity in graph representations and inter-client heterogeneity across distributed graphs. Intra-client heterogeneity leads to overfitting on individual clients, while inter-client heterogeneity complicates the aggregation of overfitted local models into a robust global model, ultimately limiting the classification capabilities of FL-GNNs. Unfortunately, existing methods fail to effectively address both challenges simultaneously, particularly in situations where label distributions and graph heterogeneity differ across clients.

To tackle the challenges presented, we proposes FasSGAT, a structure-sensitive graph attention network with label semantic embedding for federated asynchronous multi-label classification. FasSGAT is composed of three designed components: client-specific label semantics encoding tailored to each client’s label distribution, a backbone multi-label graph attention classification network that incorporates both label semantics encoding and structure-sensitive spectral features to mitigate client-side heterogeneity, and a structure-sensitive asynchronous aggregation mechanism to handle graph heterogeneity at the server level. In the client-specific label semantics encoding, FasSGAT maps labels to word vectors, and it employs a distribution-based weighting mechanism based on the label distribution of the client,. This mechanism calculates the frequency of each label occurrence on the client side, normalizes these frequencies into label distribution weights, and adjusts the word vectors accordingly. Higher-weighted word vectors indicate greater relevance to the client’s data. These weighted word vectors are then used to construct a weighted label semantic graph. To capture the label semantic contributions and correlations tied to each client’s data distribution, a multi-scale label graph autoencoder is trained on the weighted label semantic graph to extract label semantic-aware embedding features. These features are then integrated into the backbone multi-label classification network. In the backbone, FasSGAT constructs a hierarchical multi-label graph attention network, which not only incorporates label semantic-aware embedding features but also integrates structure-sensitive spectral features, representing structural invariance based on the truncated Laplacian matrix, to address the overfitting caused by client-side graph heterogeneity. On the server side, FasSGAT implements a structure-sensitive asynchronous aggregation mechanism that relies on encrypted typical graph structural invariance features from each client to aggregate them into a global model, effectively managing graph heterogeneity. Experiments on multi-label benchmarks demonstrate FasSGAT’s effectiveness compared to baselines and SOTA models. Ablation studies confirm the critical role of the 3 main components.

The main contributions of this paper are as follows:

1. The paper proposes FasSGAT, a novel approach for handling label distribution discrepancies in federated learning with graph-structured data by incorporating client-specific label semantics encoding. This method utilizes a distribution-based weighting mechanism, which adjusts label word vectors based on the label frequencies within each client’s dataset, ensuring that the model captures more relevant label semantics for each client.
2. The FasSGAT designs a backbone network that combines label semantic-aware embedding features with structure-sensitive spectral features to address intra-client and heterogeneity in graph classification tasks. Additionally, a structure-sensitive asynchronous aggregation mechanism is designed for the server to effectively aggregate client models using encrypted graph structural invariance features, addressing graph heterogeneity across clients and enabling the formation of a robust global model.

The remainder of this paper is organized as follows: Sect. "Related works" reviews related work, including learning methods for FL and multi-label learning. Section "Proposed model" introduces the proposed FasSGAT model. Section "Experiments" details the experimental set-up and validates the effectiveness of FasSGAT through experiments. Finally, Sect. "Conclusion" presents the conclusions.

## Related works

Federated learning (FL) algorithms, despite their advancements, exhibit several limitations that hinder their effectiveness in real-world scenarios. While FedAvg^1^ establishes a foundational framework through iterative local training and global aggregation, its performance degrades significantly under data heterogeneity—a common challenge arising from label imbalance (divergent class distributions across clients) or domain shift (feature distribution discrepancies despite shared labels). This misalignment between local updates and the global objective persists as a fundamental bottleneck. Recent graph-based approaches introduce their own constraints. Recent developments have introduced graph neural networks (GNNs) and graph-based federated learning frameworks to address these challenges. By representing clients or data as interconnected nodes in a graph, GNNs model structural relationships (e.g., client similarity or label correlations) to guide the aggregation process or feature alignment. For example, graph-guided aggregation refines model averaging by leveraging client topology, mitigating biases caused by label skew^3^, while cross-client feature propagation using attention mechanisms helps align heterogeneous domains^4^. These graph-based methods incorporate relational priors into FL, improving its adaptability to mismatches in data distributions. Among them, FedPIA^9^ introduces dynamic adapter permutation for personalized FL, achieving higher accuracy on non-IID medical data compared to FedAvg. FedCFA^10^ mitigates aggregation bias via counterfactual client-weighting, reducing label-skewed bias. TRAIL^11^ employs blockchain-based trust-aware scheduling to filter malicious clients while preserving privacy through zero-knowledge proofs. DCHM^12^ enables efficient collaboration between small client models and large foundation models, cutting communication costs. FedUnlearn^13^ pioneers gradient-based unlearning to comply with GDPR. These works highlight trends toward personalization (FedPIA, DCHM), trust mechanisms (TRAIL, FedCFA), and regulatory alignment (FedUnlearn). However, existing methods overlook critical aspects: First, graph structures introduce additional heterogeneity when applied to FL frameworks, particularly concerning cross-client graph heterogeneity, which significantly impacts graph classification. Second, most approaches focus on building data-to-label mappings without fully leveraging the semantic information inherent in graph labels. Multi-label semantic relationships often contain valuable structural correlations that remain underexplored. Therefore, this paper proposes a dual-perspective analysis of federated graph classification, simultaneously addressing data heterogeneity and multi-label semantic relationships to bridge these research gaps.

In multi-label federated learning, techniques like label-specific feature extraction^6,14^, nonlinear dependency modeling^15^, or multi-dimensional classification^16^ specifically address label skew and domain shifts by explicitly modeling label correlations. GNNs naturally extend these approaches by treating label dependencies as graph edges, enabling joint optimization of feature representations and label relationships. Graph federated frameworks can deploy GNNs locally to model intra-client label graphs and globally to integrate cross-client correlations, ensuring cohesive learning even when label spaces do not overlap. For example, FGCN^12^ facilitates collaborative multi-label classification by propagating label-aware embeddings across a federated graph, while warped graph learning^15^ adaptively modifies label graphs to reflect domain-specific biases. By combining graph-based relational modeling with FL’s decentralized nature, these approaches enhance robustness against both label and feature heterogeneity, setting the stage for scalable, privacy-preserving learning in interconnected, data-rich environments.

## Proposed model

**Fig. 1:**
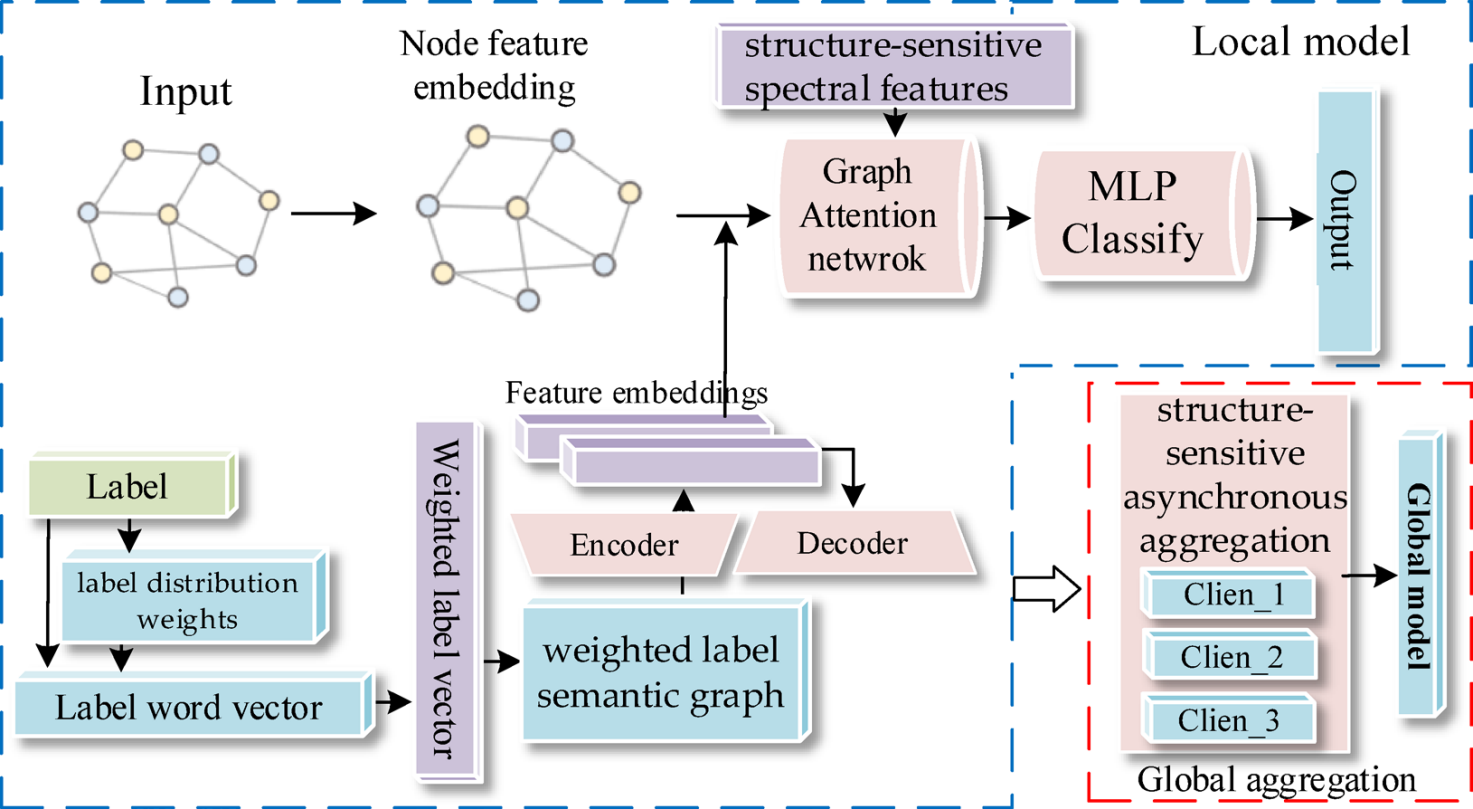
The structure of the proposed FasSGAT.

This paper proposes a structure-sensitive graph attention network with label semantic embedding for federated asynchronous multi-label classification (FasSGAT). The model structure of FasSGAT is shown in the Fig. 1:

The proposed FasSGAT algorithm follows a structured workflow to address federated asynchronous multi-label classification challenges.

1. Initially, client-specific label semantics encoding processes local label distributions by transforming labels into word vectors, normalizing label frequencies into distribution weights, and constructing weighted label semantic graphs.
2. A multi-scale label graph autoencoder then extracts semantic-aware embedding features from these graphs.
3. The backbone multi-label classification network integrates these features with structure-sensitive spectral features derived from truncated Laplacian matrices, forming a hierarchical graph attention architecture that mitigates client-side heterogeneity.
4. Finally, the server-side structure-sensitive asynchronous aggregation mechanism utilizes encrypted graph structural invariance features from clients to iteratively update a global model, effectively managing graph heterogeneity across distributed nodes.

Next, the paper details the model specifics.

### Problem formulation and setup

In this study, we assume that *K* clients participate in the federated learning process during each communication round, with each client having access to a local private graph dataset, denoted as* G* = {*G*_1_, *G*_2_, …, *G*_*K*_}. The task for each client can be framed as a standard multi-label graph classification problem. Specifically, for a graph data *x* with a corresponding label *y* = [*y*_1_,*y*_2_,…,*y*_C_], where *C* represents the total number of classes, the label *y*_*i*_ = 1 indicates that the i-th class is present in the graph, while *y*_*i*_ = 0 means it is absent. In this setting, local clients are responsible for predicting the presence or absence of each class within the graph. The overarching goal of the federated learning process is to generate a globally aggregated model that effectively handles multi-label graph classification, thereby addressing the core objective of this task.

1 $$W = \arg \mathop {\min }\limits_{W} \sum\nolimits_{i = 1}^{{M^{i} }} {\frac{{M^{i} }}{\left| M \right|}} L_{i} \left( W \right)$$

In multi-label graph FL, each client and server share the same label space with a total of C categories. However, label distributions differ across different clients and graph structures are heterogeneous in different clients. Thus, our goal is to tackle the multi-label graph FL classification task.

### The basic framework on client

On client devices, FasSGAT maintains three components: client-specific label semantics encoding tailored to each client’s label distribution, a backbone multi-label graph attention classification network that incorporates both label semantics encoding and structure-sensitive spectral features to mitigate client-side heterogeneity.

#### Data preprocessing and dual branch graph construction

For the client-specific label semantics embedding learning, FasSGAT maps labels into word vectors. Specifically, the label embeddings are defined as *L* = {*l*_1_, *l*_2_,…,*l*_*C*_}, where each *lc*$\in$*R*^*d*^ corresponds to the *c*-th class label, with *d* denoting the embedding dimension. Our framework operates through two key phases: First, we convert label information into textual representations to generate label-specific embedding features. To achieve this, we employ the CLIP vision-language model^17^ as our foundational architecture, specifically utilizing its frozen text encoder to produce stable, pre-aligned label embeddings. These CLIP-generated embeddings serve as supervisory signals during model training, capitalizing on their inherent capacity to encode semantic relationships between labels through similarity patterns. This approach effectively preserves the rich inter-label correlation information captured during CLIP’s extensive pre-training on multimodal data.

Then, based on the current client’s label distribution, FasSGAT designs an instance-level weighting mechanism. This mechanism calculates the frequency of labels occurring on the current client and normalizes these frequencies of all labels as label distribution weights for weighting the obtained label word vectors.

For the current client’s dataset, count the absolute frequency of each label. Let the client have a label set *Y* = {*y*_*1*_*,y*_*2*_*,…,y*_*K*_}, with the number of data samples being *N*, The frequency of label *y*_*k*_ is:

2 $$f_{k} = \sum\nolimits_{i = 1}^{N} {I\left( {y_{k} \in Y_{i} } \right)}$$

where, *I*(·) is the indicator function. By normalizing the frequencies into a probability distribution, the weight of each label is obtained. The weight *w*_*k*_ of label *y*_*k*_ is calculated through softmax normalization as follows:

3 $$w_{k} = \frac{{f_{k} + \varepsilon }}{{\sum\nolimits_{j = 1}^{K} {f_{j} + \varepsilon } }}$$

This label distribution weights aim to emphasize the label semantic information corresponding to the data of the current client, thereby enhancing the multi-label classification performance of the local data with client-specific label semantics learning. The word vectors with higher weights indicate that the corresponding label semantic information is more correlated with the data for the current client. After this, the label distribution weights would be used to the aforementioned label embeddings:

4 $$\tilde{l}_{k} = l_{k} * w_{k}$$

Thus, the weighted label semantic embeddings can be formulated as $\tilde{L} = \left\{ {\tilde{l}_{1} ,...,\tilde{l}_{C} } \right\}$. To model these correlation information, FasSGAT constructs a weighted label semantic graph, in which a node is a weighted label semantic embedding, and an edge is the similarity of its corresponding nodes, which can be calculated as:

5 $$edge_{i,j} = Similarity\left( {\tilde{l}_{i} ,\tilde{l}_{j} } \right) = Cosine\left( {\tilde{l}_{i} , \, \tilde{l}_{j} } \right),$$

where *Cosine*(*i*, *j*) is the correlation feature between nodes *i* and *j*. For the correlation features between nodes *i* and *j*, FasSGAT uses Cosine similarity for measurement, where $\tilde{l}_{i}$ is the weighted label semantic embedding feature data for node *i*.

To model label semantics within the weighted label semantic graph, we propose a multi-scale graph attention auto-encoder architecture that hierarchically learns relational patterns. In FasSGAT, the weighted label semantic graph serves as input to this autoencoder, where a Neural Attention Mechanism systematically extracts and parameterizes correlation features. This mechanism operates through three coordinated components: (1) a similarity evaluator that quantifies pairwise relationships among weighted label semantic embeddings, (2) attention weight generators that adaptively scale interaction intensities, and (3) multi-resolution feature aggregators that preserve both local and global dependencies. Crucially, we implement a neural similarity metric to dynamically weight feature interactions, effectively addressing the non-linear impact of varying relationship strengths in the embedding space. This function measures the similarity between two input vectors as formula 6:

6 $$\begin{aligned} Similarity_{ij} = & \,Neural\left( {A \times W \times \tilde{l}_{i} , \, A \times W \times \tilde{l}_{j} } \right) \\ & \quad = MLP\left( {Concate\left( {A \times W \times \tilde{l}_{i} , \, A \times W \times \tilde{l}_{j} } \right)} \right), \\ \end{aligned}$$

where *A* is the adjacency matrix of the masked label correlation graph and *W* is learnable weights, *SLN*() is a single-layer neural network. Based on this function, attention coefficients are defined as formula 7:

7 $$\alpha_{ij} = {\text{softmax}} \left( {Similarity_{ij} } \right) = \frac{{\exp \left( {Similarity_{ij} } \right)}}{{\sum\nolimits_{j} {\exp \left( {Similarity_{ij} } \right)} }},$$

where *α*_*ij*_ are the attention coefficients for *f*_*i*_ and *f*_*j*_. Thus, the attention feature for the current node *i* can be expressed as formula 8:

8 $$f_{i} = ReLU\left( {\sum\nolimits_{j} {\alpha_{{_{ij \in adj\_1} }} V\tilde{l}_{j} } } \right).$$

We formulate our objective as encoding attention features. These features form the foundation for our Averaged Multi-Head Attention (AMHA) architecture, which systematically aggregates attention patterns across multiple subspaces. The complete implementation of this attention block, as mathematically defined in Eq. 9, enables efficient feature integration while preserving the distinctive characteristics captured by our exponential similarity measure:

9 $$feature_{i} = ReLU\left( {\frac{1}{K}\sum\nolimits_{k = 1}^{K} {\sum\nolimits_{j} {\alpha_{{_{ij \in adj\_1} }}^{k} V^{k} \tilde{l}_{j} } } } \right)$$

Following the computation of attention features for the neighborhood adjacency matrix *adj*_1 at the finest scale, we extend our approach to calculate attention features at two additional, coarser scales. Specifically, this study considers a hierarchical framework comprising three distinct scales: 1, 2, and 3. Subsequently, we aggregate these multi-scale features to form a comprehensive encoding representation *F*. To precisely capture the numerical relationships between the electricity values and enhance forecasting accuracy, the multi-scale graph attention auto-encoder employs a reconstruction loss based on the masked label embedding graph. The loss function, as defined in Eq. 10, quantifies the difference between the reconstructed and original graph embeddings, guiding the model to learn a representation that preserves the underlying patterns in the data:

10 $$Loss\_ae = \frac{1}{N}\sum\nolimits_{n} {\left\{ {\left( {X_{n} - {\text{Recon}}_{n} } \right)^{2} } \right\}}$$

#### The backbone classification network

##### Backbone structure

The backbone used in FasSGAT is a three-layer multi-label graph self-attention neural network (ML-GAT) designed to effectively capture contextual relationships among nodes and their neighbors in graph-structured data. This model leverages a hierarchical architecture that progressively refines node representations, allowing it to focus on complex relational patterns in the graph data.

- *First Layer*: The initial layer of the network is responsible for transforming raw node features into latent embeddings. This is achieved through a linear projection with a weight matrix *W*(1), which maps the input node features into a higher-dimensional space. Following this transformation, a graph self-attention mechanism is applied to capture the interactions between nodes and their immediate neighbors. The attention mechanism allows the model to weigh the importance of each neighboring node, highlighting the most relevant connections for label prediction.
- *Second Layer*: In the second layer, the embeddings produced by the first layer undergo a refinement process through another graph self-attention operation with learnable parameters *W*(2). This layer is crucial for capturing higher-order interactions between nodes that are not immediately apparent from the raw feature data. By attending to both direct and indirect relationships among nodes, the second layer enables the model to develop a more nuanced understanding of the graph structure, allowing for better prediction of label correlations across nodes.
- *Third Layer*: The final layer maps the aggregated feature representations from the second layer to the multi-label prediction space. It does this through a sigmoid-activated output layer with a weight matrix *W*(3), which outputs a set of probabilities for each label. The sigmoid function ensures that each label is predicted independently, suitable for multi-label classification tasks. This layer effectively synthesizes all the information gathered from the previous layers to make accurate predictions.

Each layer in the ML-GAT employs the multi-head attention to enhance the model’s ability to focus on specific relational patterns across labels. Multi-head attention allows the network to compute several attention scores in parallel, each attending to different aspects of node relationships. The attention heads independently compute edge-specific coefficients, which help the model to focus on distinct, label-specific relational patterns. In each attention head, the attention score *α*_*ij*_(*l*) between two nodes *i* and *j* in layer *l* is calculated using the following formula:

11 $$\alpha_{i,j}^{\left( l \right)} = {\text{LeakyReLU}}\left( {a^{\left( l \right)} \left[ {W^{\left( l \right)} h_{i}^{\left( l \right)} ||W^{\left( l \right)} h_{j}^{\left( l \right)} } \right]} \right)$$

Here, *a*(*l*) is a learnable vector that defines the attention mechanism for layer *l*, *h*_*i*_(*l*) and *h*_*j*_(*l*) represent the node embeddings for nodes *i* and *j* at layer *l*, and || denotes concatenation. The LeakyReLU activation function is applied to introduce non-linearity and to allow for a broader range of attention values. The attention scores are then normalized across all neighboring nodes *j*$\in$*N*_*i*_ using the softmax function, ensuring that the attention coefficients sum to 1 for each node’s neighbors.

Once the attention coefficients are computed, the node representations are updated by aggregating the features of neighboring nodes, weighted by their respective attention scores. By employing these multi-layered graph self-attention, the ML-GAT backbone in FASSGAT aims to capture complex node relationships in graph-structured data for multi-label classification tasks.

##### Introducing label semantic-aware embedding features

The features are then integrated into the backbone model by concatenating the node features from the graph input data with the encoded label correlation embeddings, forming an input that is subsequently fed into the backbone model. Thus, one has

12 $$Output_{tmp} = MLP\_classifier\left( {x,F} \right),$$

where x denotes node features in the graph input data, *F* encoded label correlation embeddings features, and *Output* is the predicted logit (*MLP* denotes the multi-layer network classifier).

##### Introducing structure-sensitive spectral features

The spectral features of a graph can alleviate the graph heterogeneity problem, mainly due to their ability to mathematically abstract and uniformly express the graph structure. In this paper, we introduce the Laplacian matrix of the graph structure and truncate the large eigenvalues to extract the key information of the graph structure. This key information is used to model the core structural features of the heterogeneous graph. By incorporating these core structural features into the classification process, we mitigate the overfitting issues caused by the aggregation of graph structures across clients.

Specifically, for the *n*-th data, the client performs truncated feature decomposition on its local adjacency matrix *A*_*n*_ as follows:

- Compute the Laplacian matrix:$L_{n} = D_{n} - A_{n}$
- Select the top *M* largest eigenvalues:$\lambda 1_{n} \ge \lambda 2_{n} \ge \lambda 3_{n} ,..., \ge \lambda M_{n}$
- Corresponding eigenvectors: $$U_{n} = \left[ {u1_{n} ,u2_{n} , \cdots \,,uM_{n} } \right]$$
- Generate structure-sensitive spectral features: $g_{n}^{spectral} = Flatten\left( {U_{n} } \right)$

The structure-sensitive spectral features are introduced into the backbone for classification as Formula (13):

13 $$Output\left( n \right) = MLP\_classifier\left( {x_{n} ,F_{n} ,g_{n}^{spectral} } \right)$$

##### Training

The loss function is denoted as:

14 $$Loss = \frac{1}{N}\sum\nolimits_{n} {\left\{ {Loss\_ae + CrossEntropy\left( {label_{n} ,Output\left( n \right)} \right)} \right\}}$$

After training local models on clients, we generate a global model in the server.

### Training global models on the server

The primary objective of the federated learning process is to generate a globally aggregated model that effectively performs multi-label classification on graph data, as described in Formula (1). However, since different clients have distinct label semantics and graph structures, the models trained on these clients may prioritize these features differently. As a result, mismatches in label semantics and graph structures across clients can lead to inaccurate multi-label predictions, negatively affecting the overall performance of the federated learning system. Furthermore, inter-client heterogeneity complicates the aggregation of local models into a robust global model, ultimately limiting the classification capabilities.

Therefore, FasSGAT implements a structure-sensitive asynchronous aggregation mechanism that relies on encrypted typical graph structural invariance features from each client to aggregate them into a global model, effectively managing graph heterogeneity.

To achieve this, we first average all $g_{n}^{spectral}$ from the each client to obtain a $\tilde{g}_{k}^{spectral}$ that represents the *k*-th client’s graph structure, and then apply privacy protection by adding Gaussian noise to satisfy $\in$-differential privacy.

15 $$\tilde{g}_{k} = \tilde{g}_{k}^{spectral} + N\left( {0, \, \sigma^{2} I} \right),\quad \sigma = \frac{\Delta f}{\varepsilon }$$

where Δ*f* is the sensitivity of the eigenvectors, $\in$ is set 10 in the experiments. The encrypted typical graph structural features, along with the client model parameters, are transmitted to the server.

On the server side:

1. Spectral similarity partitioning is performed first, and a spectral similarity matrix *S* is constructed between the clients:

16 $$S_{ij} = \frac{{\tilde{g}_{i} \cdot \tilde{g}_{j} }}{{\left\| {\tilde{g}_{i} } \right\| \times \left\| {\tilde{g}_{j} } \right\|}}, \, i,j \in \left[ {1,...,K} \right]$$

where, *K* is the number of clients. Based on *S*, spectral clustering is performed to group the topologically similar clients into {G_1_,G_2_,…,G_C_}.

2) Asynchronous aggregation within groups.
  Federated Averaging (FedAvg) is applied within each group:

17 $$W_{Gp} = \frac{1}{{G_{p} }}\sum\nolimits_{{k \in G_{p} }} {W_{k} }$$

The inter-group models are fused through a gating mechanism:

18 $$W_{global} = \sum\nolimits_{p = 1}^{C} {\beta_{p} W_{Gp} } , \, \beta_{p} = \frac{{trace\left( {S_{Gp} } \right)}}{{\sum\nolimits_{q} {trace\left( {S_{Gq} } \right)} }}$$

where *S*_*Gp*_ is the intra-group similarity submatrix with Cosine similarity, and *β*_*p*_ reflects the consistency strength within the group.

### Complexity analysis for FasSGAT

The computational complexity of FasSGAT can be decomposed into two main components: local model training and global model aggregation. The local model architecture comprises three key modules: (1) label correlation modeling, (2) structure-sensitive spectral feature embedding, and (3) attention-based classification. Compared to conventional GNN-based federated learning approaches, the additional computational burden of FasSGAT primarily concentrates in the first two modules. Specifically, the label correlation modeling module involves label vector construction and correlation encoding embedding. While the former leverages pre-trained CLIP models without introducing extra computation, the latter employs a three-layer attention encoder-decoder framework with complexity denoted as *O*_*label*_(*n*). The structure-sensitive spectral feature module, though requiring Laplacian matrix computation, reduces its complexity to *O*(*m*·*nnz*) through iterative methods, where m represents iteration counts and *nnz* indicates non-zero elements. Crucially, the global aggregation process reuses precomputed spectral features from local models, incurring only marginal overhead from weight coefficient calculations. This systematic analysis demonstrates that FasSGAT’s computational overhead remains relatively modest despite its enhanced capabilities.

Next, we analyze the effectiveness of the proposed FasSGAT based on experiments.

## Experiments

Since the construction of graph representations is also part of the work presented in this paper, to validate the algorithm’s effectiveness, we utilized graph datasets and image datasets converted into graph data. To verify the effectiveness of our proposed learning framework, we conduct extensive evaluations on various benchmark datasets, including the Tox21 dataset, MUV dataset, Sider dataset, ogbg-ppa dataset, MS-COCO, and the Substation Defect Detection Dataset. Specifically, we begin by describing the datasets used in our experiments. Next, we conduct comparative experiments to verify the effectiveness of the proposed FasSGAT.

### Data set and experiment settings

We validate the performance of our method on centralized datasets partitioned artificially for FL, including Tox21 dataset, MUV dataset, Sider dataset, ogbg-ppa dataset, MSCOCO dataset, and Substation Defect Detection Dataset.

1. *The Tox21 dataset*, comprising of 11,764 training chemicals, 289 leaderboard evaluation chemicals and 633 final evaluation chemicals, was downloaded from the challenge website (National Center for Advancing Translational Sciences (NCATS), 2014) on January 9th, 2019. Features were min–max normalized to (0, 1) based on training chemicals. After data processing, the dataset comprised of 7831 graphs and 12 classes.
2. *The SIDER dataset* is a widely used resource in computational pharmacology and drug discovery for studying drug-side effect associations. The latest version (SIDER 4.1) contains data up to 2015. 1,427 unique side effects grouped into 27 System Organ Classes. Each drug is associated with multiple side effects.
3. *The ogbg-ppa dataset* is a set of undirected protein association sub graphs extracted from the protein association networks of 1,581 different species, covering 37 broad biological taxa (including mam mals, bacteroideae, archaea, etc.), including 158,100 samples.
4. *The MUV dataset* is designed to validate virtual screening methods with minimal bias, focusing on structurally diverse compounds and challenging decoys. Contains 17 targets (tasks) and 93,087 samples.
5. *The MS-COCO dataset* contains about 122,218 images containing common objects, and the standard multi-label formulation covers 80 object class annotations for each image. We transform the MS-COCO dataset into graph datasets. Convert each image in the MS-COCO dataset into graph data and transform its multi-label classification problem into a multi-label graph classification task.
6. *Substation Defect Detection Dataset* contains 8,307 images capturing substation equipment under diverse real-world conditions, annotated with 17 defect labels. These include component-level failures (e.g., blurred dial, cracked insulator, damaged cover plate), safety violations (absence of safety helmet/workwear, smoking), and system-level anomalies (abnormal system operating status, switchgear equipment anomalies).

Both image datasets are processed by first performing segmentation and then using a pre-trained ResNet model to extract features from image blocks. The cosine similarity between the features of these image blocks is calculated to construct the graph data. For compassion with the existing methods, we introduce different evaluation metrics for different datasets. The attributes and evaluation metrics are listed in Table 1.

**Table 1:** The attributes and evaluation metrics.

Datasets	Graphs	Classes	Evaluation metrics
Tox21	7831	12	ROC-AUC, F1 scores
SIDER	1427	27	ROC-AUC, F1 scores
MUV	93,087	17	ROC-AUC, F1 scores
ogbg-ppa	158,100	37	Accuracy, F1 scores
MS-COCO	122,218	80	F1 scores
Substation	8,307	17	F1 scores

Following Reference^18^, we adopt ROC-AUC as the evaluation metric for comparison, and the results presented are derived from this reference. Our experiments on the Tox21, MUV, and Sider datasets employ identical federated learning (FL) configurations. Since numerous studies validate their proposed models on ogbg-ppa using Accuracy as the evaluation metric, we also adopt this metric for consistency. For the MS-COCO and Substation Defect Detection Dataset, we adhere to the conventions established in multi-label image classification literature^8^ by employing per-class (C) and overall (O) average precision (C-AP and O-AP), precision (C-P, O-P), recall (C-R, O-R), and F1 scores (C-F1, O-F1). All experiments are implemented using PyTorch, with training conducted on a single NVIDIA RTX 4090 GPU equipped with 24GB memory. During each round of local training, we perform 10 epochs of optimization using the Adam optimizer with a learning rate of 5e-4 and a batch size of 16. For the federated learning configuration, the number of communication rounds (T) is set to 60.

### Data availability statement

The Tox21 dataset is available on (https://github.com/filipsPL/tox21_dataset), the SIDER dataset is available on (http://sideeffects.embl.de/), the ogbg-ppa dataset is available on (https://ogb.stanford.edu/docs/graphprop/), the MUV dataset is available on (http://www.pharmchem.tu-bs.de/lehre/baumann/MUV.html), the MS-COCO dataset is available on (http://cocodataset.org), and the Substation Defect Detection is available on (https://pan.baidu.com/s/1qCIGlCi54AwY0b_qX9sG2A?pwd=cuth).

### Comparison of experiments

To evaluate the performance of the proposed FasSGAT, we compare it with widely used models. The compared models contain graph attention based models. In this paper, we use 3 strategy for FL in comparisons. The FedAvg algorithm is introduced in^19^ to train the local models using local data on clients and the central server aggregates the updated model parameters through iterative model averaging. Other strategies are listed as follows:

- *FedAvg-GraphSAGE*: A FL model using GraphSAGE in clients for training. The GraphSAGE^20^ is a graph neural network model used for embedding learning of graph nodes. By sampling and aggregating the information of neighboring nodes onto the target node, it learns the representation vector of the node.
- *FedAvg-GAT*: A FedAvg model using GAT in clients for training. The GAT is a special neural network designed specifically for processing graph structured data, whose core working principle is to calculate the relationships between nodes through attention mechanisms.
- *FL-C_Tran*^8^: A FedAvg model using C_Tran in clients for training. In C_Tran, we replace the CNN with a graph attention layer.
- *FedGMTL-GraphSAGE*: A FedGMTL model using GraphSAGE in clients for training. FedGMTL is a federated learning (FL) framework that integrates Multi-Task Learning (MTL) with Federated Learning to enable collaborative training of multiple related but distinct tasks across decentralized clients while preserving data privacy.
- *FedGMTL-GAT*: A FedGMTL model using GAT in clients for training.
- *SpreadGNN-GraphSAGE*^18^: A SpreadGNN model using GraphSAGE in clients for training. SpreadGNN is a federated learning framework that can achieve model personalization and decentralized topology simultaneously.
- *SpreadGNN–GAT*^18^: A SpreadGNN model using GAT in clients for training.
- *FedLGT*^8^: FedLGT is served as a customized model update technique while exploiting the label correlation at each client. In FedLGT, we replace the CNN with a graph attention layer.

First, we test the models’ performance on the Tox21 dataset, MUV dataset, and Sider dataset. The results are shown in Table 2.

**Table 2:** Test AUC Results on Tox21 dataset, MUV dataset, and Sider dataset.

	Tox21	MUV	Sider
FedAvg-GraphSAGE	0.582	0.6578	0.582
FedAvg-GAT	0.6035	0.709	0.5857
FL-C_Tran	0.6167	0.6814	0.5973
FedGMTL-GraphSAGE	**0.6664**	0.6856	0.629
FedGMTL-GAT	0.6594	0.6899	0.61
SpreadGNN-GraphSAGE	0.585	0.703	0.5873
SpreadGNN -GAT	0.6056	0.713	0.6034
FedLGT	0.6322	0.7176	0.6119
**FasSGAT**	0.6602	**0.7331**	**0.6412**

**Fig. 2:**
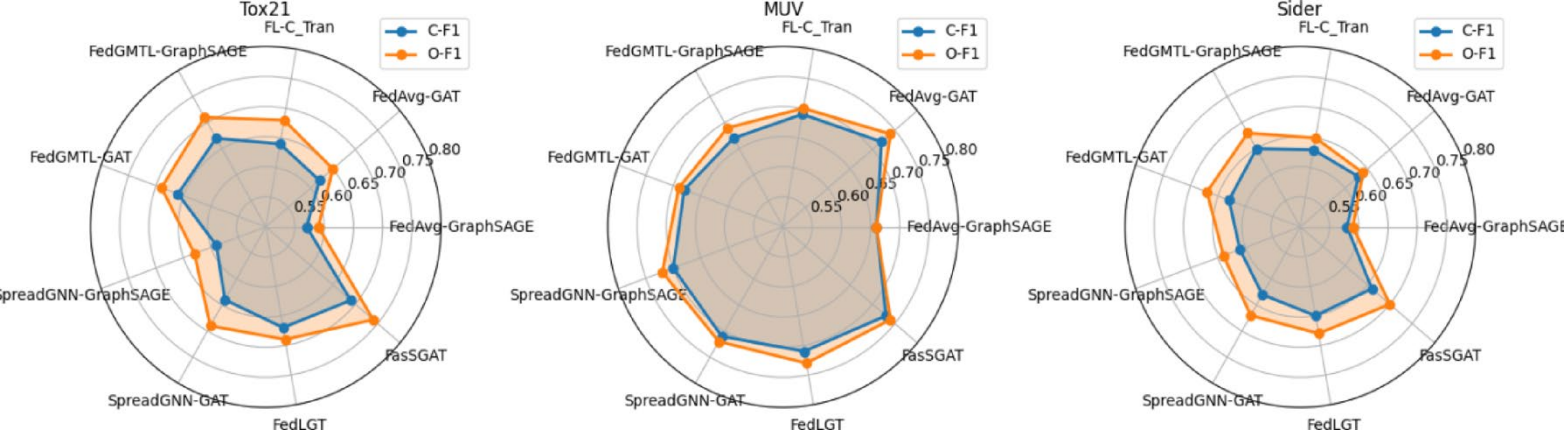
The performance of FasSGAT compared with the other models on Tox21 dataset, MUV dataset, and Sider dataset.

The test F1 scores of the 3 datasets are shown in Table 3 and Fig. 2. Significant values are in bold and underline.

As shown in the Tables 2 and 3, and Fig. 2, the experimental results demonstrate that FasSGAT achieves competitive or state-of-the-art performance across all three datasets. On Tox21, FasSGAT slightly trails the top-performing FedGMTL-GraphSAGE but outperforms all other baselines, including FedGMTL-GAT and FedLGT. For the MUV dataset, FASSGAT attains the highest ROC-AUC, surpassing FedLGT and SpreadGNN-GAT, indicating strong generalization in complex multi-task scenarios. Notably, FasSGAT delivers its most significant improvement on the Sider dataset, outperforming the previous best FedGMTL-GraphSAGE. While FedGMTL variants excel on Tox21 and Sider, FasSGAT balances performance across all tasks, achieving the highest overall average score, which suggests effective integration of global and local knowledge without overfitting to specific datasets. These experiments verify that the label embedding and the aggregation method are effective.

**Table 3:** Test F1 Scores on Tox21 dataset, MUV dataset, and Sider dataset.

	C-F1-Tox21	O-F1-Tox21	C-F1-MUV	O-F1-MUV	C-F1-Sider	O-F1-Sider
FedAvg-GraphSAGE	0.57	0.59	0.66	0.66	0.58	0.59
FedAvg-GAT	0.62	0.65	0.72	**0.74**	0.63	0.64
FL-C_Tran	0.64	0.68	0.69	0.70	0.63	0.65
FedGMTL-GraphSAGE	0.67	0.71	0.67	0.69	0.65	0.68
FedGMTL-GAT	0.66	0.69	0.68	0.69	0.63	0.67
SpreadGNN-GraphSAGE	0.59	0.63	0.7	0.72	0.61	0.64
SpreadGNN -GAT	0.64	0.69	0.71	0.72	0.63	0.67
FedLGT	0.67	0.69	0.71	0.73	0.65	0.68
**FasSGAT**	**0.69**	**0.74**	**0.73**	**0.74**	**0.66**	**0.70**

Significant values are in bold and underline.

**Fig. 3:**
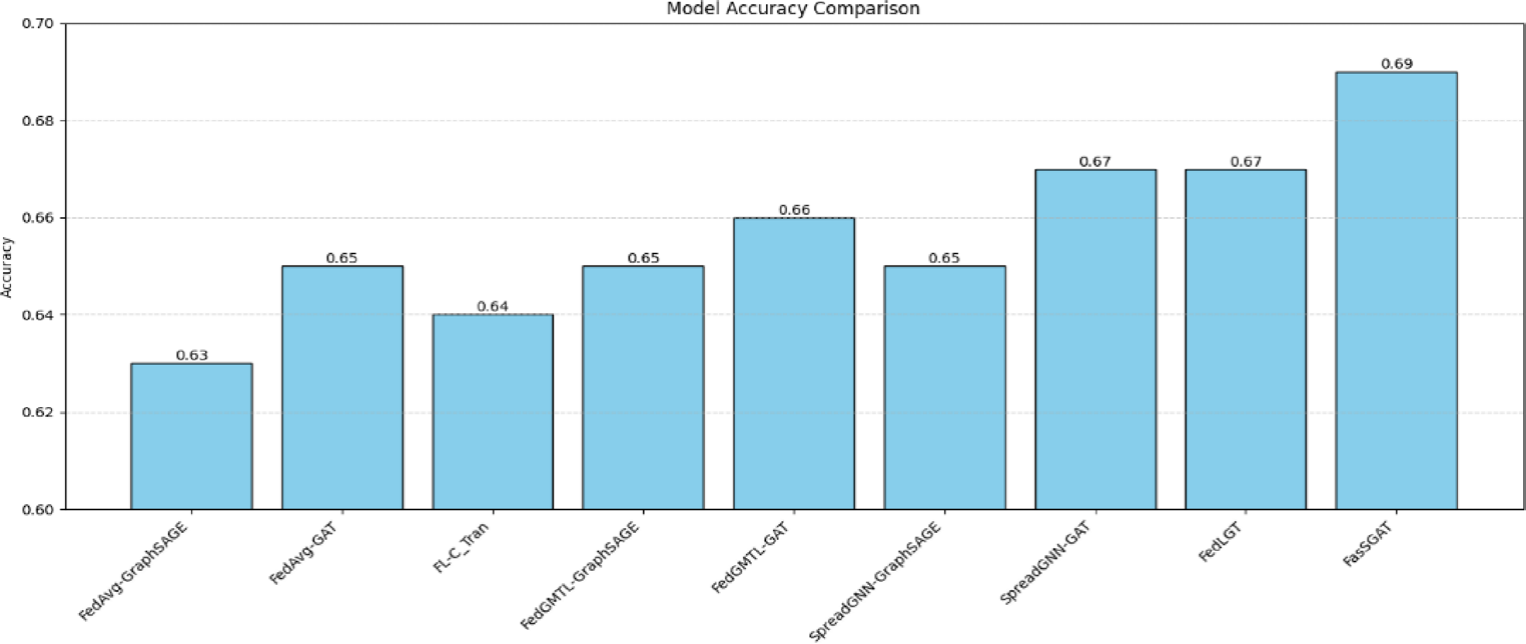
The Accuracies of FasSGAT compared with the other models on ogbg-ppa.

**Fig. 4:**
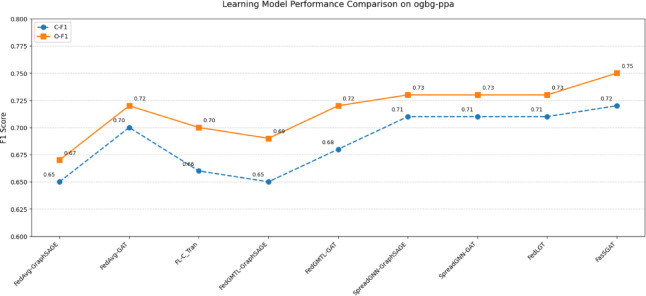
The F1 scores of FasSGAT compared with the other models on ogbg-ppa.

Next, we present the test results of the proposed FASSGAT alongside the compared models, specifically focusing on ogbg-ppa. The test Accuracy for the various models across the datasets are illustrated in Figs. 3 and 4.

As shown in Figs. 3 and 4, the experimental results indicate that FasSGAT achieves competitive performance, attaining the highest performances among the evaluated methods.

In the next experiment, we verify the effectiveness of the proposed FasSGAT on the MS-COCO dataset. The results are shown in Table 4.

**Table 4:** Results of FasSGAT compared with the commonly used models on MS-COCO dataset.

	C-P	C-R	C-F1	O-P	O-R	O-F1
FedAvg-GraphSAGE	0.739	0.656	0.695	0.781	0.712	0.745
FedAvg-GAT	0.744	0.666	0.703	0.789	0.714	0.75
FL-C_Tran	0.762	0.671	0.714	0.794	0.715	0.752
FedGMTL-GraphSAGE	0.743	0.667	0.704	0.791	0.716	0.752
FedGMTL-GAT	0.743	0.669	0.704	0.793	0.717	0.753
SpreadGNN-GraphSAGE	0.756	0.672	0.710	0.789	0.722	0.754
SpreadGNN -GAT	0.759	0.681	0.718	0.793	0.728	0.759
FedLGT	0.772	0.7	0.734	**0.802**	0.737	0.768
**FasSGAT**	**0.779**	**0.725**	**0.743**	**0.803**	**0.741**	**0.778**

Significant values are in bold and underline.

Our proposed algorithm, FasSGAT, demonstrates superior performance compared to other approaches in the MS-COCO dataset under the federated learning framework. As shown in the results, FasSGAT achieves the highest overall F1 score, tied with FedLGT, and the highest overall precision, surpassing all other methods. Additionally, FASSGAT shows competitive performance in per-class metrics. This indicates that FASSGAT not only excels in overall classification accuracy but also maintains consistent performance across individual classes.

**Fig. 5:**
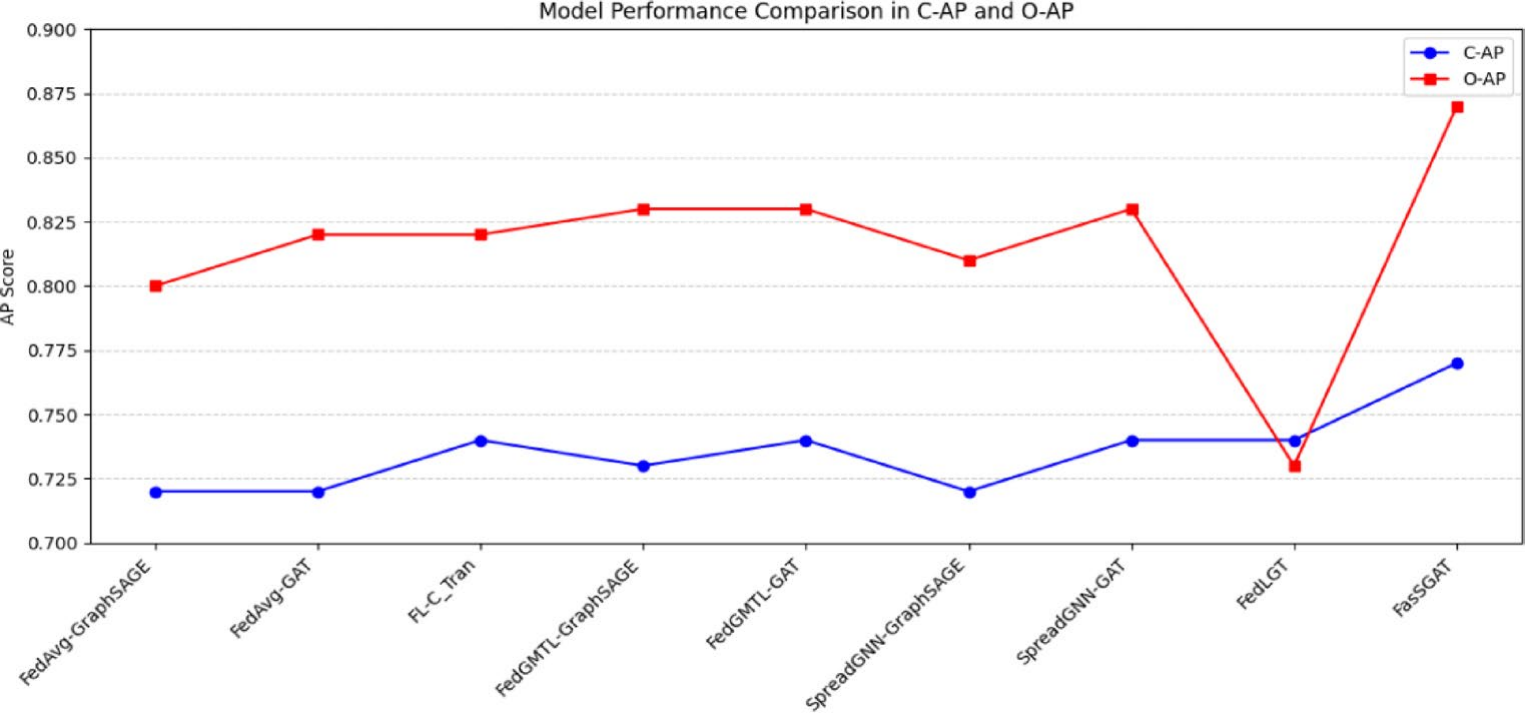
The performance of FasSGAT compared with other models on AP of MS COCO dataset.

Next, we present the test results of the proposed FasSGAT alongside the compared models, specifically focusing on AP. The test AP for the various models across the datasets are illustrated in Fig. 5.

As shown in Fig. 5, FasSGAT achieves state-of-the-art performance on the MSCOCO dataset, demonstrating superior class-level precision while maintaining competitive overall accuracy. In the next experiment, we verify the effectiveness of the proposed FasSGAT on the Substation Defect Detection dataset. The results are shown in Table 5.

**Table 5:** Results of FasSGAT compared with the commonly used models on Substation Defect Detection Dataset.

	C-P	C-R	C-F1	O-P	O-R	O-F1
FedAvg-GraphSAGE	0.639	0.606	0.622	0.754	0.703	0.728
FedAvg-GAT	0.644	0.604	0.623	0.758	0.701	0.728
FL-C_Tran	0.662	0.604	0.631	0.770	0.724	0.746
FedGMTL-GraphSAGE	0.649	0.601	0.624	0.760	0.717	0.737
FedGMTL-GAT	0.653	0.609	0.630	0.765	0.719	0.741
SpreadGNN-GraphSAGE	0.656	0.602	0.627	0.768	0.723	0.745
SpreadGNN -GAT	0.659	0.611	0.634	0.764	0.728	0.745
FedLGT	0.672	0.607	0.637	0.764	0.739	0.751
**FasSGAT**	**0.683**	**0.612**	**0.646**	**0.772**	**0.754**	**0.762**

Significant values are in bold and underline.

**Fig. 6:**
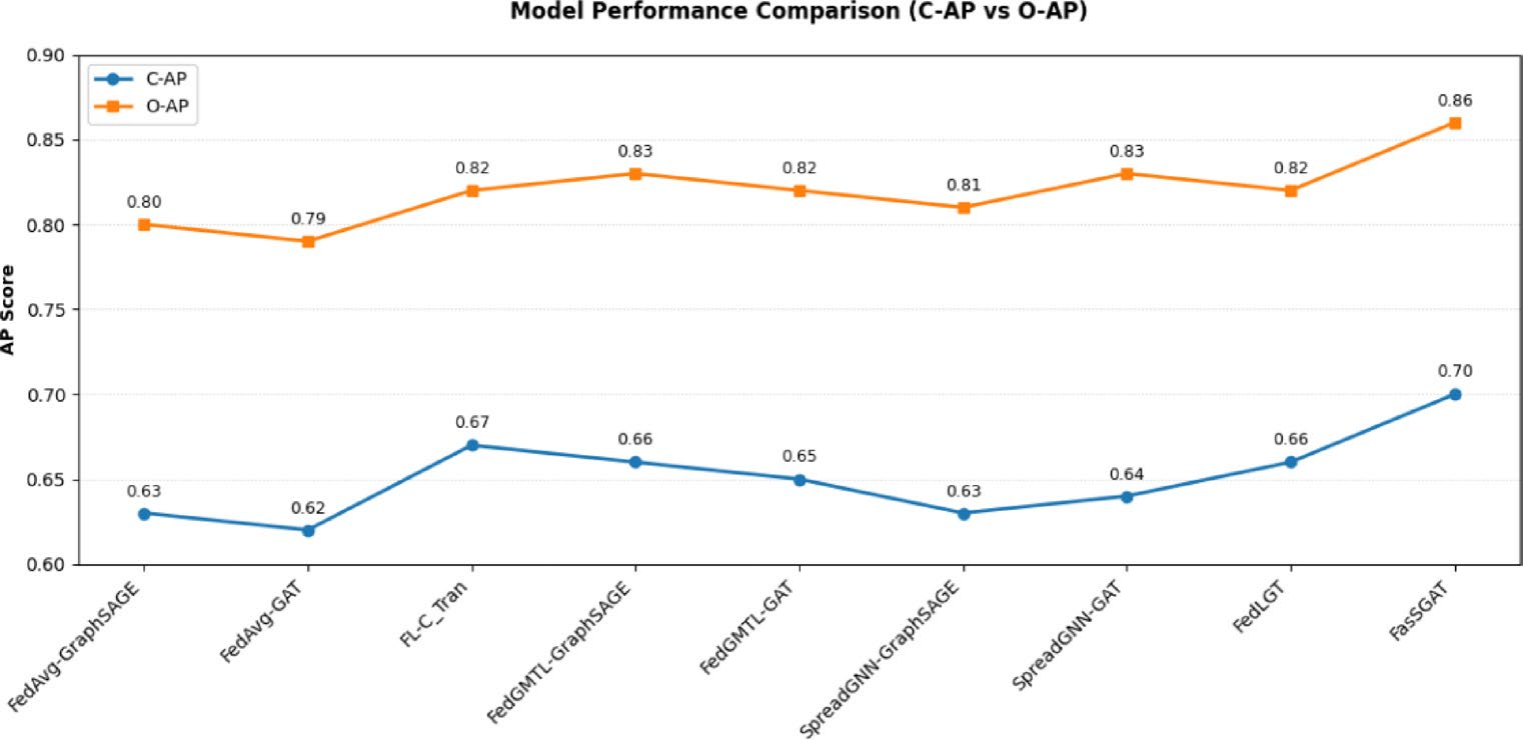
The performance of FasSGAT compared with the other models of Substation Defect Detection dataset.

FasSGAT verifies exceptional performance in the Substation Defect Detection dataset through federated learning, outperforming other algorithms across most of key metrics. With the highest F1 scores, FasSGAT exhibits better in its classifications compared to other models. Next, we present the test results for the proposed FasSGAT, comparing its performance with other models, with a particular focus on APs. The test AP values for the various models on the Substation Defect Detection dataset are shown in Fig. 6.

As illustrated in Fig. 6, the proposed FasSGAT algorithm achieves the higher AP values on the Substation Defect Detection dataset in a federated learning setting, outperforming other algorithms. This indicates that incorporating label semantic strength information into the model can effectively improve the performance of federated learning models on the Substation Defect Detection dataset.

### Ablation experiments

In this section, we perform ablation studies to highlight the contributions of the client-specific label semantics encoding and structure-sensitive asynchronous aggregation mechanism for the FasSGAT. The results are shown in Table 6.

**Table 6:** Performance of FasSGAT in the ablation experiment.

	Tox21	MUV	Sider
FasSGAT-without_weighted	0.6591	0.7033	0.6175
FasSGAT-without_graph	0. 6541	0. 7127	0. 6201
FasSGAT-without_multi-scale	0. 6611	0.7215	0.6304
FasSGAT-without_asyagg	0.6622	0.7197	0.6302
FasSGAT	**0.6637**	**0.7233**	**0.6323**

Significant values are in bold.

**Fig. 7:**
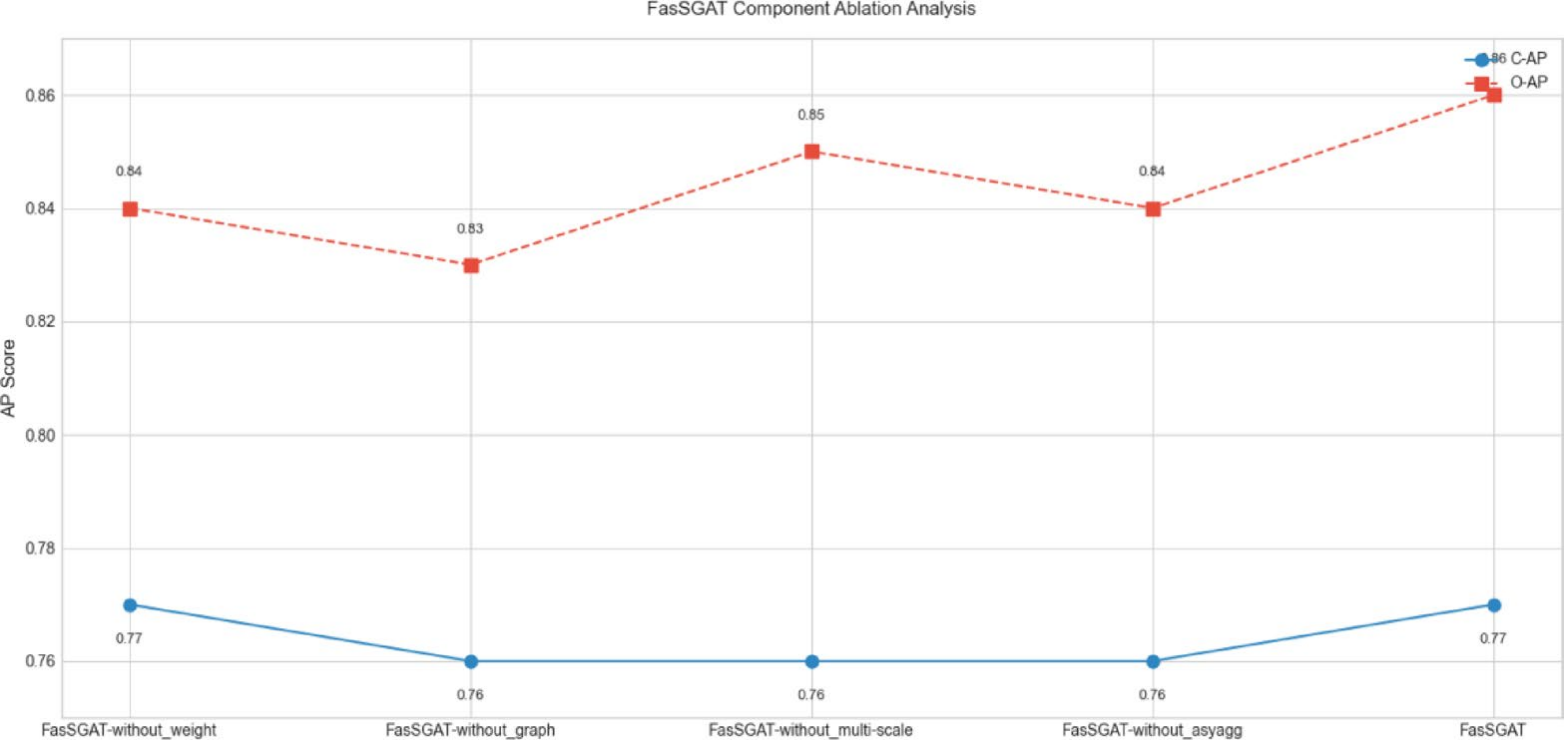
The ablation performance of FasSGAT on APs of MS COCO dataset.

In Table 5, FasSGAT-without_asyagg is the FasSGAT without using the structure-sensitive asynchronous aggregation mechanism, FasSGAT-without_multi-scale is the FasSGAT without using the multi-scale attention in the label embedding, FasSGAT-without_graph is the FasSGAT without using the label graph encoding, and FasSGAT-without_weighted is the FasSGAT without using the label weighting. As Table 5 shows, the FasSGAT performs better than FasSGAT-without_asyagg which means that the proposed adversarial-fairness aggregation for the FasSGAT is effective, FasSGAT performs better than FasSGAT-without_weighted which means that the designed state weighting operation is effective for FL, and FasSGAT performs better than FasSGAT-without_graph which means that the using multi-scale graph attention autoencoder is effective for FL. We also test the effectiveness of the label semantic graph attention framework and structure-sensitive asynchronous aggregation mechanism on MS COCO dataset in Table 7 and Fig. 7.

**Table 7:** Ablation experiments of FasSGAT on MS COCO dataset.

	C-P	C-R	C-F1	O-P	O-R	O-F1
FasSGAT-without_weighted	0.757	0.694	0.724	0.796	0.733	0.763
FasSGAT-without_graph	0.757	0.692	0.723	0.799	0.733	0.765
FasSGAT-without_multi-scale	0.762	0.693	0.726	0.797	0.732	0.763
FasSGAT-without_asyagg	0.761	0.701	0.730	0.799	0.735	0.766
FasSGAT	**0.769**	**0.715**	**0.741**	**0.802**	**0.744**	**0.772**

Significant values are in bold.

As Table 7 and Fig. 7 show, the experimental results verify the contributions of the client-specific label semantics encoding and structure-sensitive asynchronous aggregation mechanism for the FasSGAT.

### Discussions

To systematically demonstrate our algorithm’s advantages over conventional approaches, we employ a dual-pronged analytical framework. The first key innovation lies in our label correlation learning module, which was specifically designed to address graph heterogeneity by establishing correspondences between label semantics and graph structures. This module injects supervised semantic information into the graph modeling process through carefully designed label embeddings. Complementing this semantic approach, we design structure-sensitive spectral feature embedding that captures essential graph structural information via Laplacian eigenvectors. By synergistically integrating these two components—one providing semantic supervision and the other encoding structural patterns—our method effectively mitigates graph heterogeneity from both semantic and structural perspectives. Notably, the spectral features serve a dual purpose: not only do they enhance local graph representations, but we also repurpose them as weights in our aggregation mechanism to measure and alleviate inter-client graph heterogeneity. This weighted aggregation scheme, informed by the structural similarity metrics derived from spectral embeddings, constitutes our second major contribution to solving federated graph learning challenges.

The core contribution of this work lies in addressing graph data heterogeneity while enhancing classification performance in federated learning through two key innovations: label correlation modeling and structure-sensitive spectral feature embedding. While our experimental results demonstrate the algorithm’s effectiveness, we recognize that the current implementation based on graph attention networks represents just one approach among many potential graph neural network architectures. This is particularly relevant for power system applications where graph data often exhibits inherent complexity, containing both topological noise and multi-modal information that requires integrated analysis^21,22^. Moving forward, we plan to investigate more advanced neural architectures to further improve the model’s capability in handling weakly supervised learning and multi-view scenarios, thereby extending its applicability to broader real-world settings where data quality and supervision levels may vary significantly.

## Conclusion

Federated Learning (FL) offers a promising solution for privacy-preserving collaborative training, yet it faces significant challenges when applied to graph-structured data, particularly due to the non-IID distribution of data across clients. These challenges are further amplified in multi-label classification tasks and graph classification, where heterogeneity in both graph representations and label distributions can severely impact model performance. The proposed FasSGAT framework addresses these issues by integrating three key components: client-specific label semantics encoding, a multi-label graph attention network that combines label semantic embedding and structure-sensitive spectral features, and a structure-sensitive asynchronous aggregation mechanism. Through these innovations, FasSGAT effectively mitigates client-side heterogeneity and graph heterogeneity, enabling robust multi-label classification on federated graph data. Experimental results demonstrate FasSGAT’s superior performance over existing methods, with ablation studies highlighting the importance of each component in improving the overall model effectiveness. This work paves the way for more efficient and accurate FL-GNN models, particularly in scenarios involving complex, heterogeneous graph data.

## Data Availability

The Tox21 dataset is available on (https://github.com/filipsPL/tox21_dataset), the SIDER dataset is available on (http://sideeffects.embl.de/), the ogbg-ppa dataset is available on (https://ogb.stanford.edu/docs/graphprop/) , the MUV dataset is available on (http://www.pharmchem.tu-bs.de/lehre/baumann/MUV.html) , the MS-COCO dataset is available on (http://cocodataset.org) , and the Substation Defect Detection is available on (https://pan.baidu.com/s/1qCIGlCi54AwY0b_qX9sG2A?pwd=cuth) .
